# Taxane Chemotherapy for Hormone-Naïve Prostate Cancer with Its Expanding Role as Breakthrough Strategy

**DOI:** 10.3389/fonc.2015.00304

**Published:** 2016-01-11

**Authors:** Masaki Shiota, Akira Yokomizo, Masatoshi Eto

**Affiliations:** ^1^Department of Urology, Graduate School of Medical Sciences, Kyushu University, Fukuoka, Japan

**Keywords:** androgen-deprivation therapy, cabazitaxel, castration-resistant prostate cancer, docetaxel, hormone-naïve prostate cancer

## Abstract

Historically, androgen-deprivation therapy (ADT) was the only primary treatment for metastatic prostate cancer. After prostate cancer develops into castration-resistant prostate cancer (CRPC), there are a few life-prolonging drugs, including taxanes, such as docetaxel and cabazitaxel, as well as novel androgen receptor-targeting agents, such as abiraterone acetate and enzalutamide, which have been proved in clinical trials. However, the prognosis of men with CRPC is still poor. The duration from initiation of ADT to CRPC has not improved in recent decades because no novel therapeutic options have emerged. However, recently, up-front docetaxel chemotherapy has been shown to prolong progression-free as well as overall survival in men with metastatic hormone-naïve prostate cancer. This offers a new way to expand the role of chemotherapy for hormone-naïve prostate cancer. In this review, we summarize the proof-of-concept as well as the current status of taxane chemotherapy for hormone-naïve prostate cancer, focusing on phase 3 clinical trials investigating oncological outcome, and discuss the future direction in this field.

## Introduction

Previously, hormonal therapy such as androgen-deprivation therapy (ADT) using surgical or pharmacological castration and/or antiandrogens was the only gold standard for therapy of metastatic prostate cancer. However, historically, the efficacy of ADT was limited, and most prostate cancer developed into castration-resistant prostate cancer (CRPC) in about 2 years. When prostate cancer developed into CRPC, until 2004, there were no life-prolonging drugs that had been proved by clinical trial, and prognosis of men with CRPC was poor. However, in 2004, docetaxel chemotherapy was shown to prolong survival of men with metastatic CRPC (1, 2) and has become standard therapy for metastatic CRPC. Since 2010, in addition to the immunotherapeutic agent sipuleucel-T and radiopharmaceutical Ra-223, three agents for metastatic CRPC, including novel androgen receptor (AR)-targeting agents abiraterone acetate and enzalutamide, as well as novel taxane cabazitaxel, have been shown to prolong overall survival (OS) (3). Accordingly, those agents have been approved by the relevant authorities in many countries. Thus, the prognosis of men with metastatic CRPC was improved by those novel agents. However, the time to CRPC was not improved where policy dictated that only ADT should be administered and no additional therapy was considered.

Recently, some important clinical trials investigating the role of docetaxel chemotherapy for metastatic hormone-naïve prostate cancer have been reported. In this review, we summarize the proof-of-concept and the current status of chemotherapy for hormone-naïve prostate cancer, focusing on phase 3 clinical trials investigating oncological outcome, and discuss the future direction in this field.

## Proof-of-Concept by Basic Research

Although chemotherapy has been the gold standard for metastatic disease in various types of cancer, there was no life-prolonging chemotherapy for prostate cancer until 2004. However, since the emergence of docetaxel chemotherapy for metastatic CRPC in 2004 (1, 2), the hypothesis that docetaxel chemotherapy may be effective also for hormone-naïve prostate cancer was generated. Accordingly, basic research has been conducted to examine the proof-of-concept.

In 2005, Eigl et al. compared tumor growth in three groups of mice bearing Shionogi and LNCaP xenografts treated with (i) initial castration and delayed paclitaxel; (ii) initial paclitaxel and delayed castration; or (iii) simultaneous castration plus paclitaxel (4). Simultaneous ADT plus paclitaxel was more effective than sequential treatment by initial castration and delayed paclitaxel in both xenograft models (4), although there was no difference in an MDA PCa2b xenograft model (5). In line with this notion, we have recently shown cross-resistance to docetaxel in CRPC cells, but not *vice versa* using an LNCaP cell model (6). Thus, these results provide proof-of-concept for possible superior anticancer activity of up-front taxane chemotherapy for hormone-naïve prostate cancer, compared with traditional ADT followed by docetaxel chemotherapy.

Several studies have examined whether metastatic status affects the efficacy of taxane chemotherapy. Marín-Aguilera et al. reported that gene expression related to epithelial–mesenchymal transition was altered in radical prostatectomy specimens when treated with neoadjuvant docetaxel chemotherapy, and correlated with relapse (7). Also, mesenchymal marker expression was upregulated in docetaxel-resistant cells, in which knockdown of mesenchymal marker ZEB1 reversed docetaxel resistance (7). By contrast, the expression of epithelial marker E-cadherin was downregulated in docetaxel-resistant cells (8). These results suggest docetaxel resistance in highly metastatic prostate cancer with a high level of epithelial–mesenchymal transition, supporting the superior anticancer effect of docetaxel chemotherapy for non-metastatic hormone-naïve prostate cancer compared with metastatic prostate cancer.

## Metastatic Hormone-Naïve Prostate Cancer

The results of the French GETUG-AFU-15 study were reported in 2013. It did not show any improvement of OS by combining docetaxel chemotherapy with ADT for metastatic hormone-naïve prostate cancer, although docetaxel chemotherapy did improve progression-free survival (PFS) (9). The results failed to overcome the traditional concept that ADT is only useful for metastatic hormone-naïve prostate cancer. However, in 2015, the CHAARTED study conducted by the Eastern Cooperative Oncology Group investigating the utility of up-front docetaxel chemotherapy for metastatic hormone-naïve prostate cancer showed improved PFS as well as OS (10). Furthermore, the STAMPEDE trial in the UK also reported that both PFS and OS were improved by docetaxel chemotherapy in addition to ADT (11). Thus, one study failed, but two others showed significant improvement with up-front docetaxel administration for metastatic hormone-naïve prostate cancer, although all three studies were conducted with similar concept and design.

Why did those trials report controversial results? Although there seems to be several differences among those clinical trials, such as (i) the number of docetaxel cycles, (ii) sample size, (iii) proportion of high-volume disease, (iv) length of follow-up, and (v) unignorable chemotherapy-related mortality (12, 13), we suggest that the most significant difference was the subsequent treatments after progression to CRPC. At the time when the GETUG-AFU-15 trial was conducted, most novel therapeutics against CRPC were under development. Then, only a few patients were treated with cabazitaxel and only a small number of patients were recruited to clinical trials using abiraterone acetate and enzalutamide (Table 1). In contrast, the CHAARTED and STAMPEDE trials were both conducted in era of novel therapeutics for CRPC. In the CHAARTED trial, cabazitaxel was utilized more frequently (24 vs. 13%) as were abiraterone acetate and enzalutamide (44 vs. 36%), in the ADT plus docetaxel group compared with the ADT monotherapy group, although docetaxel rechallenge was less frequent (23 vs. 48%) (Table 1). Similarly, in the STAMPEDE trial, cabazitaxel was utilized more frequently (6 vs. 3%) as were abiraterone acetate and enzalutamide (35 vs. 30%) in the ADT plus docetaxel group, although docetaxel rechallenge was less (14 vs. 41%) (Table 1). Collectively, if there was no survival-prolonging agent, there may be no difference between up-front and delayed docetaxel use. However, now we can utilize several novel life-prolonging agents against CRPC, even in a postdocetaxel setting, which can be administered sequentially after up-front docetaxel chemotherapy. It has recently been reported that only 4.4% of CRPC patients in the US received AR-targeting agents and cabazitaxel in addition to docetaxel chemotherapy (14); therefore, achieving sufficient use of therapeutics for CRPC would be difficult, which may compromise the merit of novel agents.

**Table 1:** **Subsequent active therapy after progression to CRPC**.

Study	GETUG-AFU-15		CHAARTED		STAMPEDE	
Arm	ADT plus docetaxel	ADT	ADT plus docetaxel	ADT	ADT plus docetaxel	ADT
The number of enrolled cases, *n*	192	193	397	393	592	1184
The number of cases progressed to CRPC, *n*	NA	NA	238	287	311	750
Chemotherapy						
Docetaxel	54	120	54 (23%)	137 (48%)	14%	41%
Cabazitaxel	3	2	57 (24%)	37 (13%)	6%	3%
AR-targeting agent						
Abiraterone acetate	16^a^	29^a^	105 (44%)	104 (36%)	28%	23%
Enzalutamide					7%	7%
Orteronel			NA	NA	NA	NA
Others						
Sipuleucel-T	NA	NA	22 (9%)	19 (5%)	NA	NA
Ra-223	NA	NA	NA	NA	1%	0%

*^a^Referred from Ref. (12)*.

*NA, not available; percentage number represent the proportion among cases progressed to CRPC*.

Up-front docetaxel chemotherapy is effective against hormone-naïve prostate cancer, as demonstrated by a recently published meta-analysis (13). Cabazitaxel chemotherapy is also active against prostate cancer, with evidence of improved OS among men with metastatic CRPC (15). The FIRSTANA study (NCT01308567) is now examining oncological outcome of docetaxel and cabazitaxel as first-line chemotherapy for metastatic CRPC. Therefore, up-front cabazitaxel chemotherapy combined with standard ADT can also be effective for metastatic hormone-naïve prostate cancer, which has been examined by the Swedish clinical trial SensiCab (NCT01978873, Table 2). A clinical trial comparing up-front docetaxel and cabazitaxel chemotherapy combined with ADT would be intriguing.

**Table 2:** **Phase 3 clinical trials examining taxane chemotherapy for hormone-naïve prostate cancer**.

Study	Identifier	Organizer	Taxane	Stage	Control arm	Primary endpoint	Result
GETUG-AFU-15	NCT00104715	GETUG-AFU (French)	Docetaxel	Metastatic	ADT	OS, PFS	Negative
CHAARTED	NCT00309985	ECOG (US)	Docetaxel	Metastatic	ADT	OS	Positive
STAMPEDE	NCT00268476	Medical Research Council (UK)	Docetaxel	Metastatic	ADT	OS	Positive
SensiCab	NCT01978873	Örebro University (Sweden)	Cabazitaxel	Metastatic	ADT	OS	NA
SPCG 12	NCT00376792	SPCG	Docetaxel	Non-metastatic	RP (adjuvant)	PFS	NA
553	NCT00132301	Department of Veterans Affairs (US)	Docetaxel	Non-metastatic	RP (adjuvant)	PFS	NA
CALGB 90203	NCT00430183	CALGB (US)	Docetaxel	Non-metastatic	RP with neoadjuvant ADT	PFS	NA
XRP6976J 3501	NCT00283062	Sanofi	Docetaxel	Non-metastatic	RP with adjuvant vs. salvage ADT	PFS	Early terminated with negative result
RTOG-9902	NCT00004054	RTOG (US)	Paclitaxel	Non-metastatic	RT with ADT	OS	Negative
GETUG 12	NCT00055731	GETUG (French)	Docetaxel	Non-metastatic	RT (or RP) with adjuvant ADT	PFS	Improved PFS
RTOG-0521	NCT00288080	RTOG (US)	Docetaxel	Non-metastatic	RT with ADT	OS	Improved OS
SPCG 13	NCT00653848	SPCG	Docetaxel	Non-metastatic	RT with ADT	PFS	NA
05-043	NCT00116142	Dana-Farber Cancer Institute (US)	Docetaxel	Non-metastatic	RT with ADT	OS	NA
DART	NCT00651326	NCIC Clinical Trials Group (Canada)	Docetaxel	Non-metastatic	RT with ADT	PFS	NA
PEACE2	NCT01952223	Collaborative European Group	Cabazitaxel	Non-metastatic	Prostatic vs. pelvic RT with ADT	PFS	NA
SPCG 14	–	SPCG	Docetaxel	Non-metastatic, PSA recurrence after RP or RT	Bicalutamide	PFS	NA
RTOG-P-0014	NCT00030654	RTOG (US)	Docetaxel	Non-metastatic, PSA recurrence after RP or RT	Various	OS	NA
XRP6976J 3503	NCT00514917	Sanofi	Docetaxel	Non-metastatic, PSA recurrence after RP or RT	ADT	PFS	Early terminated with marginal result

*OS, overall survival; PFS, progression-free survival; RP, radical prostatectomy; RT, radiotherapy*.

## Non-Metastatic Hormone-Naïve Prostate Cancer

In addition to the promising role of taxane chemotherapy for metastatic hormone-naïve prostate cancer, there may be the possibility of efficacy for non-metastatic hormone-naïve prostate cancer. This is supported by the basic research discussed above, as well as non-randomized clinical studies showing feasibility and modest efficacy. Accordingly, several phase 3 clinical studies have been under investigation (16).

### Taxane Chemotherapy with Surgical Therapy

Radical prostatectomy for localized prostate cancer is the standard curative therapy and shows excellent oncological outcome. However, there is a high recurrence rate in high-risk patients, who are defined as high stage, high Gleason score, and high prostate-specific antigen (PSA) level at diagnosis. To improve the outcome of high-risk localized prostate cancer, the Southwest Oncology Group explored the role of mitoxantrone chemotherapy after radical prostatectomy in oncological outcome in a phase 3 clinical trial of 983 patients (SWOG S9921, NCT00004124). They failed to show significant improvement of PFS as primary endpoint (17), although mitoxantrone was palliative but not life-prolonging for men with CRPC. However, because docetaxel has been shown to be active against prostate cancer, various studies using docetaxel chemotherapy have been conducted and are in progress (Table 2) (18). The Scandinavian Prostate Cancer Group (SPCG) group has been examining whether adjuvant docetaxel for high-risk prostate cancer after radical prostatectomy can improve outcome (SPCG 12, NCT00376792). A similarly designed phase 3 trial (NCT00132301) is also ongoing in the US. A phase 3 study by the Cancer and Leukemia Group B (CALGB 90203, NCT00430183) is comparing oncological outcome after radical prostatectomy with or without neoadjuvant docetaxel chemotherapy plus ADT in 750 randomized patients with high-risk prostate cancer. So far, only one study (XRP6976J_3501, NCT00283062) has been terminated early because of poor recruitment of patients. The study was examining immediate treatment following prostatectomy vs. deferred treatment at the time of relapse with docetaxel plus ADT vs. ADT alone.

### Taxane Chemotherapy with Radiotherapy

Similarly to surgical therapy, oncological outcome of radiotherapy for high-risk prostate cancer has been modest. Although combination with ADT can improve the outcome, the improvement is far from satisfactory, and further improvement has to be pursued. The Radiation Therapy Oncology Group (RTOG) has explored the role of chemotherapy with paclitaxel, estramustine, and etoposide combined with radiotherapy and long-term ADT in a phase 3 clinical trial of 397 patients (RTOG-9902, NCT00004054). They failed to show significant improvement of OS as primary endpoint (19), although paclitaxel has never been shown to be active in prostate cancer treatment. Several clinical trials have examined the effect of active taxane chemotherapy, such as docetaxel and cabazitaxel, combined with radiotherapy as shown below and in Table 2. The GETUG 12 trial (NCT00055731) showed improved PSA decrease at 3 months, as well as improved relapse-free survival of neoadjuvant docetaxel and estramustine chemotherapy, in addition to ADT plus local therapy, mostly with definitive radiotherapy for high-risk localized prostate cancer (20, 21). Consistently, RTOG (RTOG-0521, NCT00288080) has recently reported improved OS of docetaxel chemotherapy, in addition to ADT plus radiotherapy (22). Thus, two randomized trials have recently shown superior prognosis with docetaxel chemotherapy combined with radiotherapy. In addition, The SPCG group has been investigating whether adjuvant docetaxel for high-risk prostate cancer after radiotherapy combined with ADT improves outcome (SPCG 13, NCT00653848) (23). They reported that toxicity in patients receiving adjuvant docetaxel was well tolerated although frequency of neutropenia was higher, but PFS as primary endpoint has not been reached. Similarly, they Dana-Farber Cancer Institute (NCT00116142) and a Canadian group (DART, NCT00651326) have been conducting phase 3 clinical trials examining whether docetaxel improves outcome after radiotherapy combined with ADT in patients with high-risk localized prostate cancer.

The novel taxane cabazitaxel is also under investigation. A European collaborative group has been conducting a phase 3 trial in patients with localized prostate cancer with high risk of relapse (PEACE2, NCT01952223), comparing ADT + prostate radiotherapy, ADT + pelvic radiotherapy, cabazitaxel + ADT + prostate radiotherapy, and cabazitaxel + ADT + pelvic radiotherapy.

### Taxane Chemotherapy for Non-Metastatic Recurrent Disease

For biochemical recurrent non-metastatic disease after radical prostatectomy or radiotherapy, ADT has been the primary standard therapeutic option. However, most recur in a castration-resistant fashion, requiring improvement of management of such patients.

Accordingly, the SPCG group has been conducting a phase 3 clinical trial (SPCG 14) to examine whether additional docetaxel chemotherapy improves the outcome when combined with standard ADT. Similarly, the RTOG group has been conducting a phase 3 trial (RTOG-P-0014, NCT00030654) examining the effect of immediate vs. delayed docetaxel chemotherapy with ADT for PSA recurrence after definitive therapy. ADT with or without docetaxel for patients with rising PSA after radical prostatectomy was investigated in a phase 3 trial (XRP6976J_3503, NCT00514917), but this study was terminated after all participants had completed treatment with docetaxel, showing marginal prolonged PFS by combining with docetaxel chemotherapy (19).

## Conclusion

The battle between clonal selection and adaption theories in treatment resistance is old, but it is a new debate in cancer biology. In favor of clonal selection theory, up-front docetaxel chemotherapy combined with ADT can almost eradicate androgen-dependent and CRPC cells, and result in prolonged cancer control. In contrast, possibly more in favor of adaption theory, up-front docetaxel chemotherapy can exert a superior anticancer effect compared with when it is prescribed after obtaining castration resistance. Thus, although it remains unresolved, the success of the CHAARTED and STAMPEDE trials is a major advance in this field and indicates the superiority of up-front docetaxel chemotherapy.

Thus, up-front docetaxel could be an attractive option for selected patients. However, appropriate selection of patients for up-front chemotherapy would be important since monitoring the efficacy of up-front chemotherapy is almost impossible and toxicity of chemotherapy cannot be ignored. It is important to identify patients who enjoy the benefit of up-front chemotherapy for metastatic hormone-naïve prostate cancer and establish personalized medicine. These aims could be achieved by identifying predictors of poor response to hormonal therapy or good response to chemotherapy, using clinical characteristics or serum/genetic biomarkers.

Evidence that chemotherapy can improve the oncological outcome of definitive therapy for non-metastatic prostate cancer has not been in the process of being obtained. Thus, efforts to extend the merit of up-front chemotherapy for metastatic hormone-naïve prostate cancer by applying it to non-metastatic hormone-naïve prostate cancer should be maintained. In addition, novel AR-targeting agents abiraterone acetate and enzalutamide have also been investigated in up-front application for metastatic hormone-naïve prostate cancer combined with conventional ADT, as well as non-metastatic hormone-naïve prostate cancer with definitive therapy. If those agents can improve prognosis, head-to-head comparison between up-front chemotherapy and up-front novel AR-targeting agents would be warranted in future, which could lead to major changes in prostate cancer therapy.

## Author Contributions

MS wrote the manuscript. AY and ME supervised.

## Conflict of Interest Statement

Masaki Shiota and Akira Yokomizo have received lecture fees from Janssen Pharma (Masaki Shiota and Akira Yokomizo), Astellas Pharma (Akira Yokomizo), and Sanofi (Masaki Shiota and Akira Yokomizo). Masatoshi Eto has received honoraria as the lecture moderator from Janssen Pharma, Astellas Pharma, and Sanofi.
